# Long-term post-trial follow-up of participants in randomised trials: lessons learned from the mrc / bhf heart protection study (HPS)

**DOI:** 10.1186/1745-6215-14-S1-O84

**Published:** 2013-11-29

**Authors:** Theingi Aung, Richard Bulbulia, Louise Bowman, Jane Armitage

**Affiliations:** 1 Clinical Trial Service Unit (CTSU), University of Oxford, Oxford, UK

## Introduction

Treatment effects may be significantly underestimated by analyses restricted to the relatively brief intervention phase of a randomised trial, and effects on cancer may only emerge during prolonged follow-up. In HPS, post-trial follow-up for non-fatal events was largely achieved via annual postal questionnaires, and the methods used may help inform the design of subsequent similar studies.

## Methods

20,536 patients at increased vascular risk were randomly allocated 40 mg simvastatin or placebo for a mean "in-trial" duration of 5.3 years. Post-trial follow-up of all 17,519 surviving participants yielded a mean total follow-up of 11.0 years, with non-fatal events and statin use reported by participants in annual mailed questionnaires or via GPs, supplemented with cause-specific mortality and site-specific cancer incidence via central registries.

## Results

Response rates to annual postal questionnaires were around 80% each year and total 34,555 non-fatal events were reported. Number of events reported from different sources showed: 24,691 from questionnaire, 6162 from cancer registries, 3400 via GPs, 239 from letters/phone calls and 63 from non-fatal events on death certificate. Based on these large numbers of fatal and non-fatal events, long-term follow-up of HPS reliably demonstrates the long-term efficacy and safety of lipid-lowering statin therapy.

## Conclusion

Capturing non-fatal post-trial events via postal questionnaires is effective, and allows reliable assessment of the benefits and potential hazards of the intervention being studied. However, the process was labour-intensive, and follow-up via Hospital Episode Statistics (HES) data may be an alternative cost effective means of comprehensively gathering such data in future studies.

